# Coffee consumption and alertness: a study among office workers in Jakarta

**DOI:** 10.3389/fnut.2024.1425707

**Published:** 2024-11-08

**Authors:** Herqutanto, Tria Rosemiarti, Dian Kusuma Dewi, Dewi Sumaryani Soemarko, Ari Fahrial Syam

**Affiliations:** ^1^Department of Community Medicine, Faculty of Medicine, Universitas Indonesia, Jakarta, Indonesia; ^2^Division of Gastroenterology, Department of Internal Medicine, Faculty of Medicine, Universitas Indonesia, Jakarta, Indonesia

**Keywords:** coffee, alertness, reaction time, caffeine, office workers

## Abstract

**Background:**

Coffee is widely consumed by office workers, primarily for its caffeine content, which plays an important role in improving alertness. For white-collar workers, alertness is crucial to maintaining productivity, and one measurable parameter of alertness is reaction time. Changes in reaction time can be classified as either shorter or longer compared to the initial measurement taken before coffee consumption, with a longer reaction indicating a decrease in alertness. The objective of this study is to investigate the relationship between coffee consumption and improvements in alertness.

**Materials and methods:**

This cross-sectional study compared coffee drinkers and non-coffee drinkers, assessing their alertness using the Lakassidaya tool and collecting data on coffee consumption and caffeine intake through a 7-day fluid diary. Alertness was measured in the morning (baseline) and 30 min after coffee consumption (end line). Study participants were office workers from a company in Jakarta, Indonesia.

**Results:**

A total of 121 participants completed the study, with 47.1% (*n* = 57) of them being coffee drinkers. No significant differences were found in the characteristics of the respondents. The average daily coffee consumption among these workers was 247 (157–391) mL/day, with a caffeine intake of 72 (36–121) mg/day, which was below the minimum amount typically required to trigger a positive alertness reaction. There was no significant association between coffee consumption and alertness (OR = 1.538, 95% CI: 0.288–1.467, *p* = 0.403), nor was there a significant difference in the change in reaction time between coffee drinkers and non-coffee drinkers (17.3 [1.4–32.2] ms vs. 13.0 [−3.9–26.0] ms, *p*-value = 0.111). However, there was a trend toward improved alertness in coffee drinkers, as indicated by shorter reaction times at baseline (180 [160–195.2] ms) compared to post-consumption (155 [146.6–170.2] ms).

**Conclusion:**

There was no significant association between coffee consumption and alertness improvement between coffee drinkers and non-coffee drinkers among office workers in Jakarta, Indonesia.

## 1 Introduction

Coffee is one of the most popular beverages, particularly among young adults, due to the wide variety of choices available. Its consumption has been steadily increasing year after year (1, 2). Coffee has become even more visible with the proliferation of coffee shops in office areas and the availability of vending machines. The number of coffee drinkers has been growing at an average rate of 1.1% per year. In 2017, global coffee consumption was 9,682,620 tons, which increased to 10,109,460 tons in 2018. In Indonesia, average coffee consumption was 1.4 kg/year, making it the sixth largest consumer globally, following Europe, the USA, Brazil, Japan, and Russia (3, 4). Among white-collar office workers, approximately 46.2% were reported to be regular coffee drinkers, consuming coffee approximately 3–4 times per day (5).

Ratnasari et al. showed that 40% of coffee consumers in their study were employees (5). The frequency of coffee consumption is determined by various factors. Samoggia and Riedel (6), in their literature review, classified these factors into five main categories: personal preferences, economic attributes, product attributes, consumption context, and sociodemographic factors (1, 6).

The caffeine in coffee is an active ingredient that is often considered a psychostimulant that affects the central nervous system (7). It improves cognitive function, concentration, and alertness, especially in people who experience fatigue and low focus. Caffeine has an antagonistic effect on adenosine receptors, which play a key role in regulating alertness (8). Additionally, Choi pointed out that people consume coffee not only for alertness but also for its taste, mood-boosting effects, social interaction aspects, perceived health advantages, and habitual use, indicating that alertness is a primary motivation for coffee consumption (9).

In occupational health, alertness is defined as the ability to maintain focus on a task over a period of time (10). It involves the process of receiving and maintaining sensitivity to incoming stimuli (11). For office workers, alertness is crucial for task productivity (12). One of the key parameters used to represent alertness is reaction time (13–19). Changes in reaction time can be classified as either shorter or longer, with longer reaction times indicating a decrease in alertness (20).

Previous studies (21) have reported that caffeine intake improves alertness and productivity, for instance, by preventing burnout (21–24). A review also reported that caffeine, consumed both in capsules and in coffee, could improve alertness in normal or low-alertness states (7). In Indonesia, there is limited information on the effect of coffee consumption on alertness, especially among office workers. The objective of the study was to observe the difference in alertness between coffee drinkers and non-coffee drinkers among office workers in a company in Jakarta. We hypothesize that workers who frequently consume coffee will exhibit higher alertness compared to those who do not.

## 2 Materials and methods

This cross-sectional study assessed the relationship between coffee consumption and changes in alertness among office workers in a company in Jakarta, Indonesia. Data collection was conducted in November 2023. The sample size was calculated using a proportion difference formula (Equation 1), with the proportion of coffee drinkers set at 63.6% (4, 25). The formula is shown below, applying a 10% margin of error and a 95% confidence level to ensure the precision and reliability of the results, while considering the feasibility of sample collection (26). A total of 135 subjects were initially recruited to participate in the study, with an anticipated dropout rate of 10%.


(1) $$\begin{array}{l} \mathrm{Proportion difference to calculate sample size}:\\ \begin{array}{c} n1n2={\left[\frac{\left(Za\sqrt{2PQ}+zb\sqrt{p1q1+p2q2}\right)}{\left(p1-p2\right)}\right]}^{2}\\ n1=\frac{1.96+0.84}{0.298}\\ n1={\left[\frac{1.3785+0.5634}{0.298}\right]}^{2}\\ n1=n2=57 \end{array} \end{array}$$

**Figure 1:**
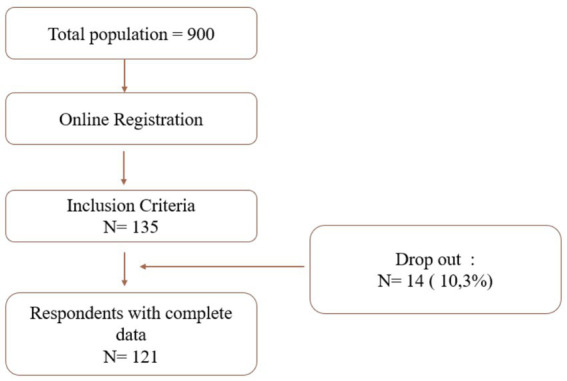
Respondent recruitment.

Figure 1 shows the recruitment process. Of the 900 potential participants who registered, 135 participants met the eligibility criteria. However, a dropout rate of approximately 10.3% (*N* = 14) due to incomplete data reduced the final sample size to 121 respondents.

The majority of the participants had an administrative job with a sedentary lifestyle. This study was approved by the Research Ethics Committee of the Faculty of Medicine Universitas Indonesia—Dr. Cipto Mangunkusumo General Hospital (FKUI-RSCM) under protocol number 23-10-1651 and conducted in accordance with the Declaration of Helsinki. Recruitment information was shared online through a link that directed participants to a Google Form accessible only to the researchers. Employees who met the criteria could participate in the study by providing informed consent, which was available in the link before the screening form section.

The screening form included characteristics such as age, sex, smoking status, and hours worked. Smoking status was categorized as either “smoking” or “non-smoking,” and the age category was divided into “35 and younger” and “older than 35.” The age of 35 was eliminated due to the perception that 35-year-old workers were considered less productive (27). According to Deary and Der (28), there is a steady increase in reaction time between the ages of 30 and 60, which implies a reduction in response to incoming stimulation. Body mass index (BMI) and blood pressure information were obtained from annual medical examinations.

Eligibility criteria for the study included being employed by the selected company, consenting to participate, and not taking medications such as amphetamines, antidepressants, or caffeine-containing drugs. Eligible participants were then contacted to complete a 7-day fluid diary and take an alertness test. Participants were classified into two groups: coffee drinkers and non-coffee drinkers. This classification was based on a self-administered questionnaire in which participants were asked about their coffee consumption habits. Coffee drinkers were defined as those who consumed coffee daily, while non-coffee drinkers were defined as those who did not consume coffee.

Alertness was measured by assessing the reaction time using the Lakassidaya tool, which evaluates the time required for a subject to react to the trigger given by the tool digitally (29). The Lakassidaya L77 is commonly used to measure work fatigue and alertness by recording the reaction time stimuli and is effective in assessing levels of fatigue in various settings. This tool has been used in studies involving nurses and industrial workers to determine the impact of work conditions on fatigue levels (29–32). Participants in the study were required to click a mouse to stop the running time each time the trigger was activated. The time was shown in milliseconds. The reaction time was measured twice: initially in the morning (baseline) and 30 min after coffee consumption (endline). For coffee drinkers, the endline measurement was taken 30 min after consuming coffee. For non-coffee drinkers, it was taken 30 min after the baseline measurement, without coffee consumption.

The difference between reaction times at baseline and endline was reported as the change in reaction time (25). Reaction times were then categorized as shorter or longer compared to baseline. A longer reaction time indicated a decrease in alertness. On the contrary, a shorter reaction time indicated an improvement in alertness.

Coffee consumption was measured using a 7-day fluid diary, which is considered the gold standard for assessing water and beverage intake. The validity of a 7-day fluid diary was studied by Bardosono et al. in comparison to a 24-h food recall where the 24-h food recall was underestimated (33). The assessment was self-reported, and the participants were instructed to record each water or beverage they consumed immediately after consumption. The diary was then submitted via Google Forms, which participants could access whenever needed. The participants were required to record the type of beverage and coffee they consumed, the type of glass or bottle they could pick from the options, and the serving size.

Types of beverages included in the study were water, coffee, tea, milk and derivatives, sugar-sweetened beverages, and others. For coffee, the variations included espresso, Americano, brewed coffee, instant coffee, and other coffee. “Other coffee” referred to types of coffee that contained additional liquids such as milk, chocolate, foam, and other condiments. This coffee classification was further analyzed to determine the caffeine content (34). The calculation was provided by Van Dam et al. (34), who indicated that caffeine content varies by serving size. One serving of espresso, Americano, and brewed coffee contained 63 mg, 150 mg, and 92 mg of caffeine, respectively. For other coffee types, they were treated as being equivalent to one serving size of espresso, as they were espresso-based.

The difference between the two groups was tested using the Wilcoxon test for normally distributed data or the Mann–Whitney test for non-normally distributed data. A Chi-square analysis was used to compare the differences in proportions between groups. A *p*-value of <0.05 was considered statistically significant.

## 3 Results

At the end of the study, 121 participants were included, as they had completed all the study procedures. There were no significant differences in coffee consumption habits based on sex, age, working hours, smoking status, and blood pressure between coffee drinkers and non-coffee drinkers (Table 1). Approximately 47.1% of the participants were coffee drinkers, and 49.6% were over 35 years of age. Overweight participants were 2.3 times more likely to be coffee drinkers compared to those with a normal BMI.

**Table 1:** Respondent characteristics and coffee consumption habits.

Characteristic	Coffee drinker *n*(%)	Non-coffee drinker *n*(%)	Total, *n*(%)	OR (CI 95%)	*p*-Value
Age (Med (Q1–Q3))	38 (28–43)	34 (27–45)	35 (27–44)		0.651^a^
≤ 35 years	28 (49.1)	33 (51.6)	61 (50.4)	0.907	0.789
> 35 years	29 (50.9)	31 (48.4)	60 (49.6)	(0.444–1.852)	
Sex					
Female	38 (66.7)	46 (71.9)	84 (69.4)	0.783	0.535
Male	19 (33.3)	18 (28.1)	37 (30.6)	(0.361–1.698)	
BMI (Med (Q1–Q3))	26.0 (22.8–28.6)	24.2 (21.3–27.8)	25.0 (21.8–28.3)		0.098^a^
Underweight	3 (5.3)	4 (6.3)	7 (5.8)	1.500 (0.288–7.807)	0.629
Normal	12 (21.1)	24 (37.5)	36 (29.8)	*Reference	1.000
Overweight^1^	42 (73.7)	36 (56.3)	78 (64.5)	0.429 (0.188–0.977)	0.041
Hours worked					
8 h	56 (98.2)	61 (95.3)	117 (96.7)	2.754 (0.278–27.253)	0.368
> 8 h	1 (1.8)	3 (4.7)	4 (3.3)		
Smoking status					
No	53 (93.0)	62 (96.9)	115 (95,0)	0.427 (0.075–2.427)	0.325
Yes	4 (7.0)	2 (3.1)	6 (5.0)		
Blood pressure					
Normal	49 (86.0)	55 (85.9)	104 (86.0)	1.002 (0.359–2.800)	0.997
Pre-hypertension	8 (14.0)	9 (14.1)	17 (14.0)		
Total	57 (47.1%)	64 (52.9%)	121 (100.0%)		

The alphabet letter (^a^) indicates that the analyses used the Mann–Whitney test to observe differences between groups (coffee vs. non-coffee drinkers).

There was a significant difference in caffeine intake between older and younger participants (9 mg vs. 30 mg, *p* < 0.05). Additionally, daily coffee consumption was significantly higher in overweight participants compared to those with normal BMI and underweight participants (114 [0–304] mL vs. 0 [0–154] mL vs. 0 [0–154] mL, *p* < 0.020). Overweight participants also had significantly higher caffeine intake and coffee consumption frequency. Smokers had significantly higher coffee consumption (38 [0–216] mL vs. 299 [165–481] mL, *p* < 0.05), caffeine intake (9 [0–38] mg vs. 30 [0–114] mg, *p* < 0.05), and coffee consumption frequency (0.1 [0–1.0] times/day vs. 1.4 [0.9–1.8] times/day, *p* < 0.04) compared to non-smokers (see Table 2).

**Table 2:** Respondent characteristics and coffee consumption, caffeine intake, and frequency of consumption.

Characteristic	Coffee consumption (mL)	Caffeine intake (mg)	Coffee consumption frequency (times)
Coffee consumption habit			
Coffee drinker	247 (157–391)	72 (36–121)	1.1 (0.7–1.4)
Non-coffee drinker	0 (0–0)	0 (0–0)	0.0 (0.0–0.0)
*p*-Value	0.000	0.000	0.000
Age			
≤35 year	25 (0–194)	9 (0–38)	0.1 (0.0–0.8)
>35 year	142 (0–320)	30 (0–114)	0.5 (0.0–1.3)
*p*-Value	0.072	0.037	0.055
Sex			
Female	38 (0–197)	17 (0–63)	0.2 (0.0–1.0)
Male	91 (0–350)	26 (0–142)	0.4 (0.0–1.4)
*p*-Value	0.179	0.154	0.158
BMI			
Underweight	0 (0–154)^a,b^	0 (0–34)^a^	0.0 (0.0–0.8)^a,b^
Normal	0 (0–154) ^a^	0 (0–50)^a^	0.0 (0.0–0.6)^a^
Overweight	114 (0–304)^b^	0 (27–108)^b^	0.5 (0.0–1.1)^b^
*p*-Value	0.020*	0.011*	0.034*
Hours worked			
8 h	39 (0–238)	18 (0–72)	0.2 (0.0–1.1)
>8 h	112 (16–199)	89 (8–162)	0.4 (0.0–1.0)
*p*-Value	0.832	0.237	0.904
Smoking status			
No	38 (0–216)	17 (0–70)	0.1 (0.0–1.0)
Yes	299 (165–481)	134 (87–197)	1.4 (0.9–1.8)
*p*-Value	0.014	0.004	0.004
Blood pressure			
Normal	38 (0–227)	17 (0–72)	1.1 (0.0–1.0)
Pre-hypertension	157 (0–269)	27 (0–100)	1.2 (0.7–1.1)
*p*-Value	0.648	0.569	0.583
Total	42 (0–225)	18 (0–72)	0.2 (0.0–1.1)

The asterisk sign (*) indicates a statistically significant difference. Different lowercase letters in BMI characterization showed statistically significant differences. Data are presented as median (Q1–Q3) and analyzed using the Mann–Whitney test between groups due to its data distribution.

**Figure 2:**
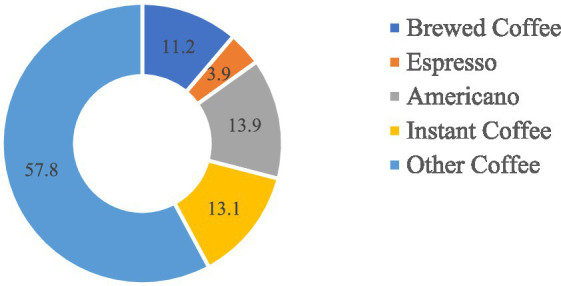
Types of coffee. Data are presented as percentages.

The most common type of coffee consumed by coffee drinkers was found to be “other coffee” (57.8%), followed by Americano (13.9%), instant coffee (13.1%), and espresso (3.9%) (Figure 2). “Other coffee” referred to types of coffee that contained additional liquids such as milk, chocolate, foam, and other condiments and was assumed to be espresso-based.

**Figure 3:**
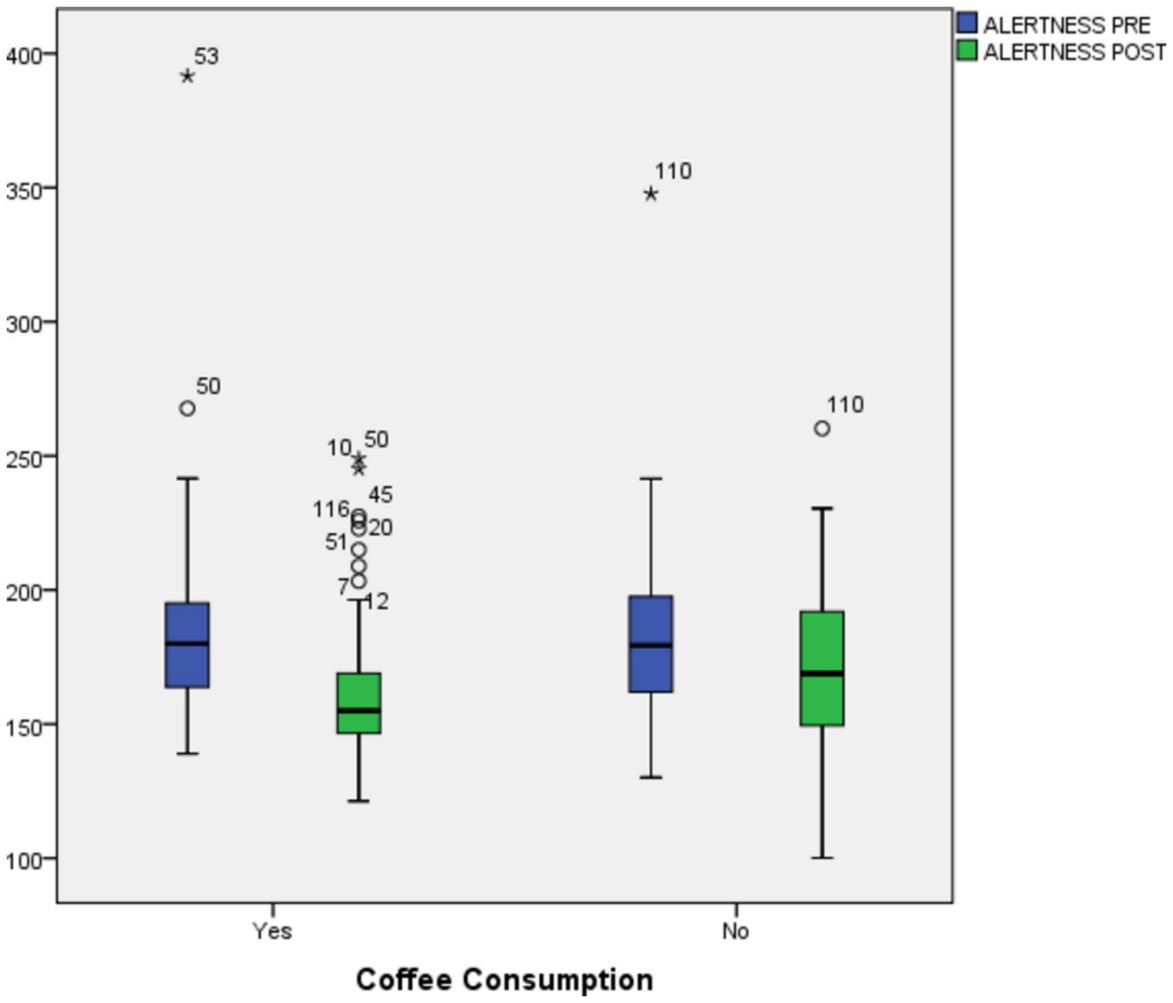
Change in reaction time between groups of coffee drinkers before and after intervention.

There was no significant difference between the two groups (180 [160.2–195.2 ms] vs. 179.3 [161.6–197.6 ms]; *p*-value = 0.990) at baseline (pre-test) and endline (post-test) (155.0 [146.0–170.2] vs. 168.8 [149.1–194.1 ms]; *p*-value = 0.069). There was a slightly bigger reduction in reaction time for the coffee drinkers between the pre-test and post-test (17.3 [1.4–32.2 ms], *p*-value = 0.000 vs. 12.9 [−3.8–26.0 ms], *p*-value = 0.000) (Table 3). Figure 3 shows a boxplot diagram of the change in reaction time between the pre-test (shown in a blue box) and post-test (shown in a green box) in both the coffee drinker and non-coffee drinker groups. Despite the insignificance compared to the non-coffee drinkers, the improvement in reaction time was more evident in the coffee drinkers group.

**Table 3:** Change in reaction time between before and after drinking.

Alertness	Coffee drinker (*n* = 57)	Non-coffee drinker (*n* = 64)	*p*-Value
Pre-test	180.0 (160.2–195.2)	179.3 (161.6–197.6)	0.990^b^
Post-test	155 (146.6–170.2)	168.8 (149.1–194.1)	0.069^b^
Change	17.3 (1.4–32.2)	12.9 (−3.8–26.0)	0.111^b^
*p*-Value	0.000^a^	0.000^a^	

Change	*n* (%)	*n* (%)	OR (CI 95%)	*p*-Value
Shorter	44 (77.2)	44 (68.8)	1.538	0.403^c^
Longer	13 (22.8)	20 (31.3)	(0.288–1.467)	

Data are presented as median (Q1–Q3). The alphabet letter (^a^) indicates that the analyses used the Wilcoxon test to observe the difference between times (pre-test vs. post-test). The alphabet letter (^b^) indicates that the analyses used the Mann–Whitney test to observe differences between groups (coffee vs. non-coffee drinkers). The alphabet letter (^c^) indicates that the analyses used the chi-squared test to observe group differences.

## 4 Discussion

A total of 47.1% of the participants consumed coffee on a daily basis. There were no characteristic differences associated with coffee consumption. However, there were differences in coffee consumption among those who were overweight and those who were smokers compared to their counterparts.

Overweight participants were 2.3 times more likely to consume higher amounts of coffee and caffeine compared to those with normal BMI. This was supported by Lee et al. (35), who found that women with higher coffee consumption tended to have a higher BMI. One study also reported that 69.6% of coffee drinkers chose to add milk to their coffee (36). In line with research conducted by Hurdawaty et al. (37), the most common types of coffee consumed were coffee with milk (48.0%) and cappuccino (20.0%). This may be due to the tendency to follow trends and preferences for certain flavors (37).

However, adding milk and sugar to coffee increases caloric intake.

However, BMI did not significantly affect alertness, as caffeine distribution increases in overweight subjects, while its half-life decreases (38). This may explain why individuals with a higher BMI do not always exhibit the effects of caffeine intake.

This study revealed a strong correlation between smoking and coffee consumption. Similar to the findings of Bjørngaard et al. (39), increased smoking frequency is directly associated with higher coffee intake, likely due to smokers’ faster caffeine metabolism. Smokers often develop a tolerance to nicotine, which may require them to consume higher doses to achieve the desired effects. This phenomenon may also occur with caffeine, causing smokers to require more caffeine to experience its stimulating effects (40). Interestingly, the benefits of higher coffee intake differ between smokers and non-smokers. Grosso et al. (41) reported in their meta-analysis that coffee consumption was associated with a reduced risk of all-cause, cardiovascular disease (CVD), and cancer mortality in non-smokers. However, the results for smokers were contradictory, likely due to the modifying effects of smoking habits (41).

There was no significant difference in alertness between coffee drinkers and non-coffee drinkers. In this study, the average caffeine intake among coffee drinkers was 72 (36–121) mg, with an average frequency of coffee consumption of 1.1 times/day. This pattern is similar to the European Food Safety Authority (EFSA), where caffeine consumption of 75–150 mg improves alertness and concentration (42–44).

This amount was lower than the caffeine intake reported in other studies to achieve the expected level of alertness. Previous studies have shown that the amount of caffeine intake needed to achieve alertness changes significantly. Doyle et al. (45) reported that the optimal caffeine intake to improve performance and alertness is approximately 6 mg/kg of body weight (45).

Additionally, Haghighatdoost et al. (46) and Pagar et al. (42) found that an optimal intake of 250 mg of caffeine, irrespective of body weight, led to significant changes in alertness (42, 46). Moderate doses of caffeine (40–200 mg) can enhance alertness and decrease reaction time. The EFSA suggests that adults can safely consume up to 400 mg of caffeine. However, exceeding this limit may lead to adverse effects such as anxiety, insomnia, sleep disturbance, increased urination, and dehydration (47).

The cross-sectional design of this study may have contributed to the differences in results compared to earlier experimental studies (43, 48–50). While the timing of the post-test—30 to 45 min after coffee consumption—was consistent with previous studies, the amount of coffee consumed by participants in this study was not controlled. This contrasts with earlier studies in which coffee or caffeine was administered to participants as a controlled intervention. The lack of control over coffee intake in this study may have influenced the results.

The lack of a significant difference in reaction time between the pre-test and post-test among coffee drinkers may be attributed to caffeine tolerance. Caffeine tolerance can develop when someone consistently consumes coffee or other caffeine-containing products. As tolerance builds, the effects of caffeine may diminish, requiring higher doses to achieve the same level of stimulation (51). This phenomenon was demonstrated in a study by Bell and McLellan (52), where the same caffeine dose provided to both coffee drinkers and non-coffee drinkers resulted in better and longer-lasting effects in the non-coffee drinkers.

As the researchers acknowledge, this study was the first to examine the association between coffee consumption and alertness improvement by measuring reaction time before and after coffee consumption using non-coffee drinkers as a comparison group among office workers in Indonesia. This study also used a 7-day fluid diary to assess coffee consumption, which is regarded as the gold standard for assessing water and beverage intake, despite its online application for the convenience of the participants.

The self-administered method, in which participants self-reported their coffee consumption, may raise concerns about accuracy. To this, the study employed enumerators to assist and remind participants to record their intake accurately. However, a limitation was that this study did not account for the type of coffee beans consumed, which may have contributed to the insignificant difference observed between coffee drinkers and non-coffee drinkers. Future studies could address this by investigating the specific coffee beans consumed by participants or by selecting sample groups more likely to consume homogeneous coffee types. An experimental study is also recommended for future research to further refine the findings.

Future research should aim for larger participant samples to increase generalizability, allowing the results to be applied to broader groups of office workers. Additionally, the methodology could be improved by shifting toward an experimental study. Including more variables to account for potential confounding factors is also recommended, as they were not fully accounted for in this study. Future studies could incorporate multivariate analyses to determine the adjusted odds ratio (OR) for the association between coffee consumption and alertness. Moreover, assessing work productivity as a primary outcome would provide valuable insights for occupational health evaluations. Other factors, such as the availability of coffee in the workplace and its impact on mental health, should also be considered, as coffee consumption has been associated with mood improvement, stress reduction, and protection from burnout.

## 5 Conclusion

There was no significant difference in changes in alertness between coffee drinkers and non-coffee drinkers before and after coffee consumption. This outcome may be attributed to the caffeine intake not reaching the minimum threshold required to produce optimal alertness effects.

## Data Availability

The original contributions presented in the study are included in the article/supplementary material further inquiries can be directed to the corresponding author.
